# Retraction Note: Decreased miR-154 expression and its clinical significance in human colorectal cancer

**DOI:** 10.1186/s12957-019-1647-z

**Published:** 2019-06-14

**Authors:** Yang Kai, Cheng Qiang, Pan Xinxin, Zhou Miaomiao, Lin Kuailu

**Affiliations:** 1 Department of Surgical Oncology, The First Affiliated Hospital of Wenzhou Medical College, Wenzhou, 325000 China; 2 Department of neurology, Huai’an No. 2 Hospital, Huai’an, Jiangsu Province China

**Retraction note: World J Surg Oncol**

**https://doi.org/10.1186/s12957-015-0607-5**

The Editor-in-Chief is retracting this article [1] due to overlap with the following articles (amongst others) [2–6].

None of the authors have responded to any correspondence from the Editor-in-Chief or publisher about this retraction.
